# Metabolic and coagulation effects of citrate: down to the last detail!

**DOI:** 10.1186/s13054-015-1154-8

**Published:** 2015-12-12

**Authors:** Patrick M. Honore, Rita Jacobs, Inne Hendrickx, Elisabeth De Waele, Viola Van Gorp, Herbert D. Spapen

**Affiliations:** ICU Department, Universitair Ziekenhuis Brussel, Vrije Universiteit Brussel, 1090 Brussels, Belgium

The recently published Liver Citrate Anticoagulation Threshold (L-CAT) trial convincingly showed that continuous renal replacement therapy with regional citrate anticoagulation (CRRT-RCA) was safe and effective in patients with severely impaired liver function. Striking findings were a relatively low incidence of acid–base disorders and a markedly long 72-h filter survival [1]. Nonetheless, we would like to comment on two important metabolic issues. First, the low (2 %) incidence of severe metabolic alkalosis, defined as a pH >7.55, is probably highly underestimated. Indeed, we recently evaluated the occurrence of metabolic alkalosis defined either as a pH >7.50 or as an apparent strong ion difference (SIDa) >45 mmol/L according to the Stewart-Figge methodology [2] in patients undergoing CRRT-RCA with an isonatremic low-chloride containing diluted citrate solution. Calculating SIDa revealed a ninefold increase in the percentage of metabolic alkalosis [3]! Accordingly, applying the SIDa approach likely will uncover a substantially higher incidence of severe metabolic alkalosis in the L-CAT patients. Second, the L-CAT investigators attributed the long filter lifespan to a more automated fine-tuning of citrate and calcium dosing. However, we recently demonstrated that keeping post-filter ionized calcium (iCa) within tight limits (i.e., 0.2–0.3 mmol/L) during CRRT-RCA resulted in a 72-h filter survival comparable with that observed in the L-CAT trial [4]. Post-filter iCa levels in all L-CAT trial groups were within the same range and thus may have accounted for a better preserved filter patency.

## Abbreviations

CRRT: continuous renal replacement therapy
CRRT-RCA: continuous renal replacement therapy with regional citrate anticoagulation
CVVH: continuous veno-venous hemofiltration
iCa: ionized calcium
L-CAT: Liver Citrate Anticoagulation Threshold
RCA: regional citrate anticoagulation
SIDa: apparent strong ion difference
