# Supplementary material for: Metabolic and coagulation effects of citrate: down to the last detail!
Source: Crit Care. 2015 Dec 12;19:433. doi: 10.1186/s13054-015-1154-8 (PMC4699326; doi:10.1186/s13054-015-1154-8)
Supplement: Related texts

# Reply: Authors’ response

**Authors:** Torsten Slowinski, Detlef Kindgen-Milles

We agree with Honoré et al. that the use of the Stewart-Figge approach probably would have discovered a higher incidence of alkalosis in the study population of the L-CAT study [1]. However, in clinical practice as well as in other studies investigating RCA the Henderson and Hasselbalch approach is used. For practical reasons in this multicenter trial and to allow better comparisons with other studies, we decided to use the latter approach in the L-CAT study.

The major outcome parameter for safety in the L-CAT study was the incidence of severe acidosis (pH ≤7.2) or alkalosis (pH ≥7.5) in patients with different degrees of liver failure. Most important, we found no significant difference between these groups and this was true not only for pH, but also for bicarbonate concentration. Thus, even if the overall incidence of alkalosis might have been slightly different using the Stewart-Figge approach, most probably no differences between the study groups would have been detected this way.

Regarding the problem of metabolic alkalosis during RCA, it is of note that other colleagues, i.e., Oudemanns-van Straaten et al. [5], reported a significantly higher incidence of metabolic alkalosis (bicarbonate >30 mmol/L) in patients treated with conventional bicarbonate containing solutions in systemic anticoagulated continuous veno-venous hemofiltration (CVVH; 26 %) compared with those who were treated with RCA-CVVH (9 %). This observation clearly shows an urgent need to analyze different protocols in CRRT with regard to their efficiency to control acid–base state.

Of note, we agree that keeping ionized calcium concentrations within the extracorporeal circuit in tight limits is the most important task in terms of avoiding filter clotting in RCA-CRRT. In vitro data showed that iCa has to be lowered to at least <0.4 mmol/L to provide sufficient anticoagulation [6].

Discussing filter lifetime as efficacy endpoint, the L-CAT study showed a filter patency rate of >90 % after 72 hours if treatment stops not caused by clotting were censored. Thus, the filter clotting rate has decreased continuously compared with earlier publications of Morgera et al. [7] and Kalb et al. [8], which used exactly the same RCA protocol. Obviously, with increasing experience and training of staff, clotting events can nowadays be avoided almost completely. This way, safe and effective CRRT can be applied and delivery failure is avoidable.
